# Infected Subgaleal Hematoma After Head Trauma in an Infant

**DOI:** 10.7759/cureus.85691

**Published:** 2025-06-10

**Authors:** Amer Salman, Thaer S Abohamra, Sania Shahid

**Affiliations:** 1 Pediatric Emergency Medicine, Al Jalila Children’s Specialty Hospital, Dubai, ARE

**Keywords:** complicated head trauma, fever after head trauma, head trauma in infants, infected scalp swelling, infected subgaleal hematoma

## Abstract

Head trauma is common among infants and children, with most cases being minor. Head trauma may be associated with long-term disability and even death, depending on the severity and the extent of brain injury. Infants and children with apparently minor injuries may be affected by clinically significant traumatic brain injury. Subgaleal hematoma is the result of an accumulation of blood in the subgaleal space following a minor head trauma. Here, we report the case of a five-month-old male who appeared initially well after minor head trauma but later developed worsening of his symptoms, including irritability, increased scalp swelling, and fever. Clinically, he was irritable with no neurological deficit, and blood investigations showed very high inflammatory markers. The clinical picture was suggestive of an infected subgaleal hematoma, so he required admission and treatment with intravenous antibiotics for seven days. Upon discharge, the patient was clinically well and had a normal clinical examination. A follow-up appointment after one month revealed a normal neurological examination.

## Introduction

Subgaleal hematoma can be caused by the application of tangential and radial forces to the scalp in minor head trauma [1,2]. The condition that results from the tearing of emissary veins and usually crosses the suture lines [3,4] is a known complication of instrumental delivery at birth. The incidence of subgaleal hematoma in neonates is approximately 0.5 per 1,000 births [5]. In moderate and severe cases of subgaleal hematoma, progressive anemia associated with hypovolemic shock can result in death, as seen in 11.8%-25% of cases [6,7]. Subgaleal hematoma can also occur after blunt head trauma in infants and older children, and even in cases of traumatic hair pulling, as seen in one reported case of hair pulling by an adult [8].

Infection can occur as a complication of subgaleal hematoma. Infection of a subgaleal hematoma is extremely rare, particularly when there is no disruption of the skin barrier. Subgaleal abscess formation without an overlying wound is rarely reported [9]. The hematoma can extend into the orbit, causing visual deficit, proptosis, ophthalmoplegia, and corneal ulceration [10-12]. The symptoms of subgaleal hematoma usually resolve within a few days to weeks. The drainage of subgaleal hematoma is rarely needed. Laboratory workup may be required in some children when an inherited or acquired bleeding disorder is suspected [13-16].

## Case presentation

A five-month-old male preterm born at 34 weeks of gestation fell from a height of approximately one meter, after which he had no vomiting, loss of consciousness, seizure, or significant scalp swelling. A day later, the infant developed irritability and fever, followed by swelling over the right side of his head, which was slowly increasing toward the occipital area.

The patient presented to the emergency department three days after the trauma and was clinically febrile (39.3°C), with tachycardia (heart rate: 183 beats/minute, respiratory rate: 46 breaths/minute, blood pressure: 106/69 mmHg, SpO_2_: 97%). He was irritable with no neurological deficit. Swelling was noted in the right parietal area extending to the occipital area with overlying tenderness, skin erythema, and skin warmth. His anterior fontanelle was flat, his pupils were reactive and symmetric, and the rest of the examination was unremarkable.

Blood investigations showed high inflammatory markers (white blood cell (WBC): 46,900 × 10^9^/L, absolute neutrophil: 33,770 × 10^9^/L, C-reactive protein: 263.4 mg/L, procalcitonin: 2.9 ng/mL, hemoglobin: 10.6 g/dL, prothrombin time: 16.9 seconds, international normalized ratio: 1.29, activated partial thromboplastin time: 45.3 seconds) (Table 1).

**Table 1:** Blood investigation results on admission. WBC: white blood cells; PT: prothrombin time; INR: international normalized ratio; aPTT: activated partial thromboplastin time

Blood investigation	Results	Reference range
WBC	46.900 × 10^9^/L	6–18.0 × 10^9^/ L
Absolute neutrophils	33.770 × 10^9^/L	1–6 × 10^9^/L
C-reactive protein	263.4 mg/L	0–5 mg/L
Procalcitonin	2.9 ng/mL	<0.5 ng/mL
Hemoglobin	10.6 g/dL	11.1–14.1 g/dL
PT	16.9 seconds	11.5–15.3 seconds
INR	1.29	0.86–1.22
aPTT	45.3 seconds	35.1–46.3 seconds

Peripheral blood smear examination showed severe leukocytosis with absolute neutrophilia and lymphocytosis. The patient’s respiratory viral screening panel, blood culture, and urine culture were negative. Lumbar puncture was traumatic and showed a WBC count of 56 × 10^6^/L (neutrophils: 43%, lymphocytes: 30%, monocytes: 27%) and a red blood cell (RBC) count of 7,000 × 10^6^/L. Screening for meningitis/encephalitis panel and cerebrospinal fluid culture were negative (Table 2).

**Table 2:** Cerebrospinal fluid test results. WBC: white blood cells; RBC: red blood cells

Cerebrospinal fluid test parameter	Result	Reference range	Interpretation
Color	Bloody	Colorless	Traumatic lumbar puncture
Appearance	Slightly turbid	Clear	Traumatic lumbar puncture
WBC	56 × 10^6^/L	0–7 × 10^6^/L	High (traumatic lumbar puncture)
Differential count	Neutrophils: 43%; lymphocytes: 30%; monocytes: 27%	-	-
RBC	7,000 × 10^6^/L	0–5 × 10^6^/L	High (traumatic lumbar puncture)
Meningitis/Encephalitis panel	Negative	Negative	-
Culture	No growth isolated after five days	No growth after five days	Normal

**Figure 1:**
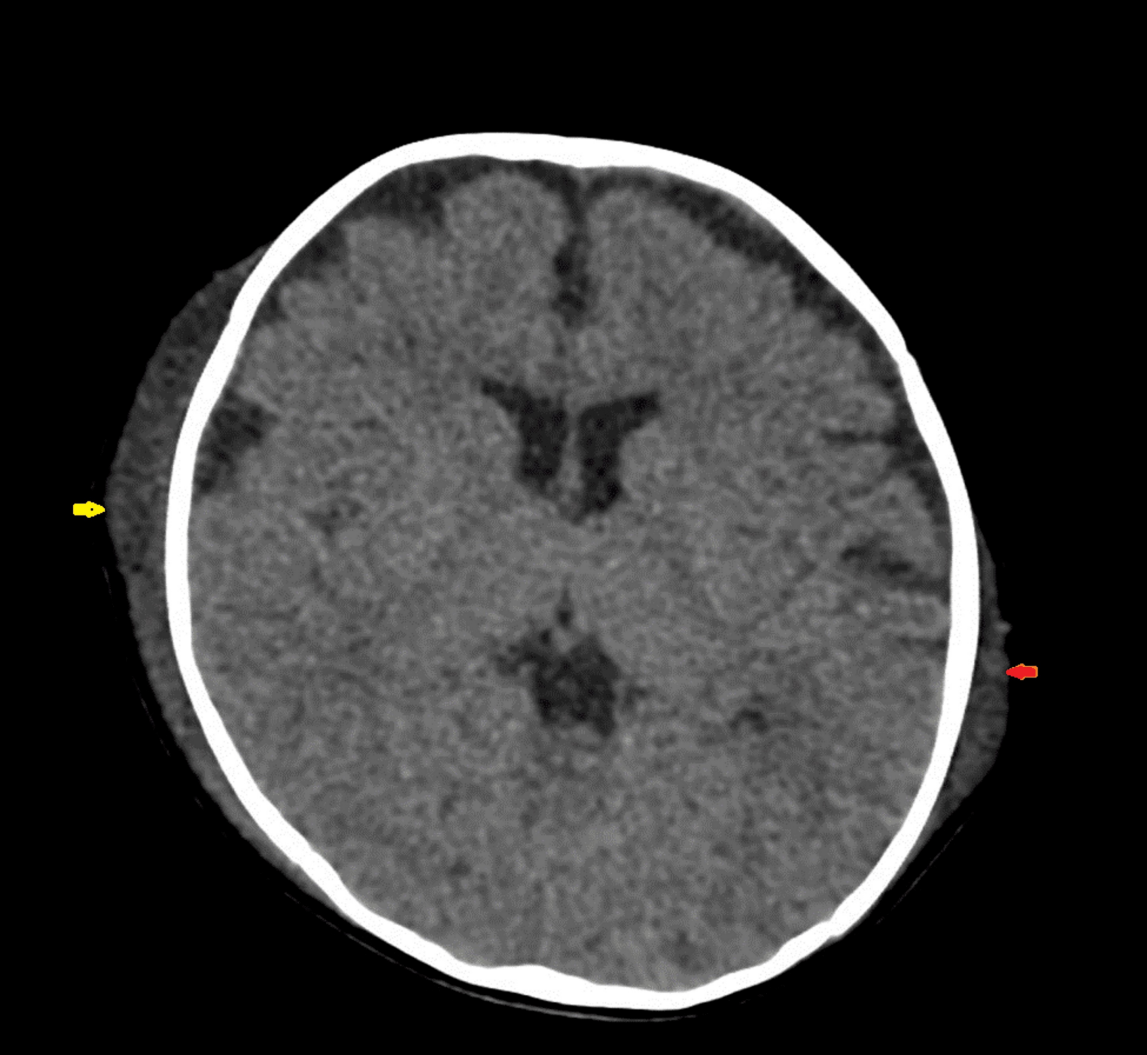
CT of the brain (axial plane) showing right-sided subgaleal hematoma (yellow arrow) with involvement of the opposite side (red arrow).

**Figure 2:**
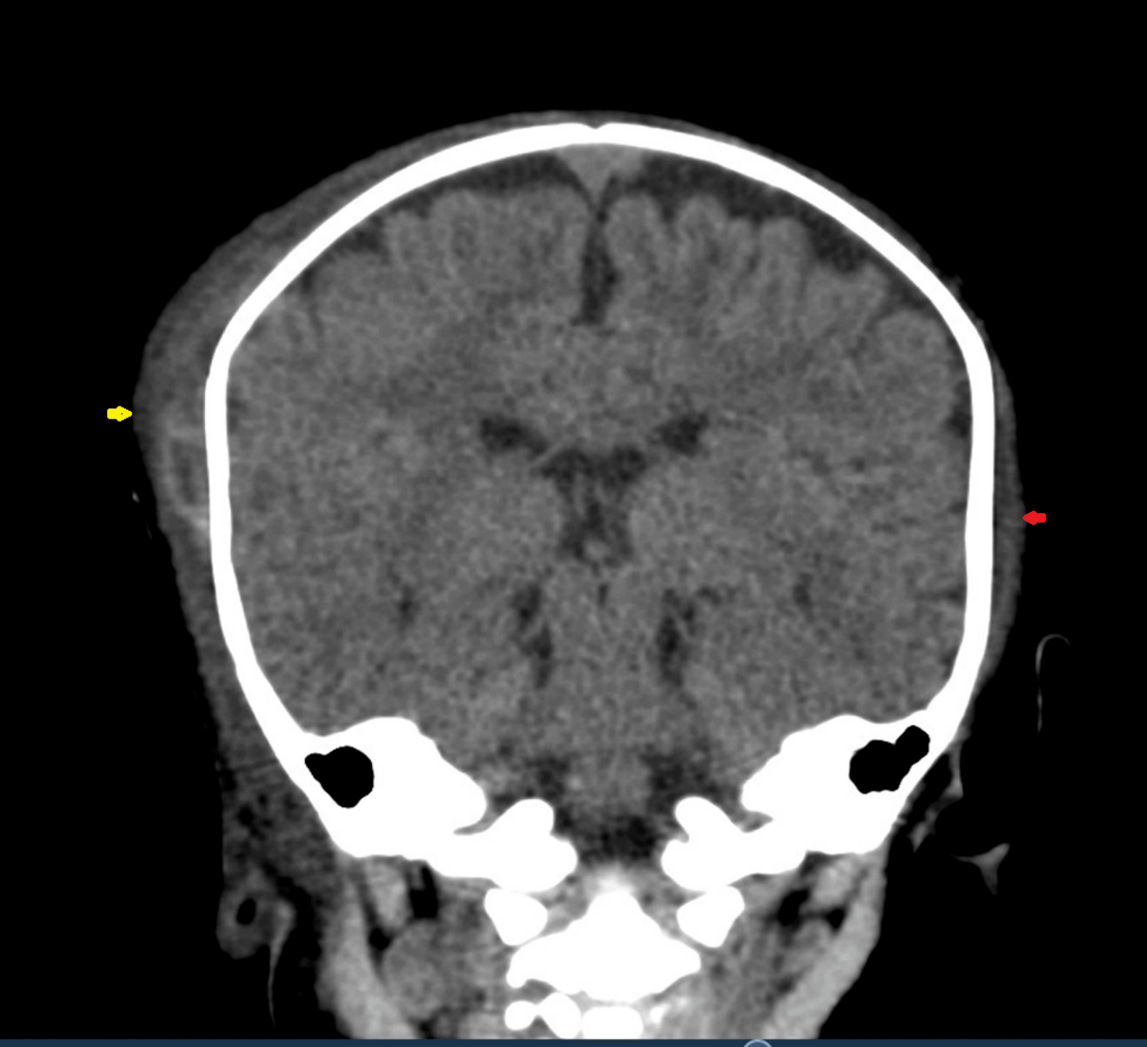
CT of the brain (coronal plane) showing right-sided subgaleal hematoma (yellow arrow) with involvement of the opposite side (red arrow).

**Figure 3:**
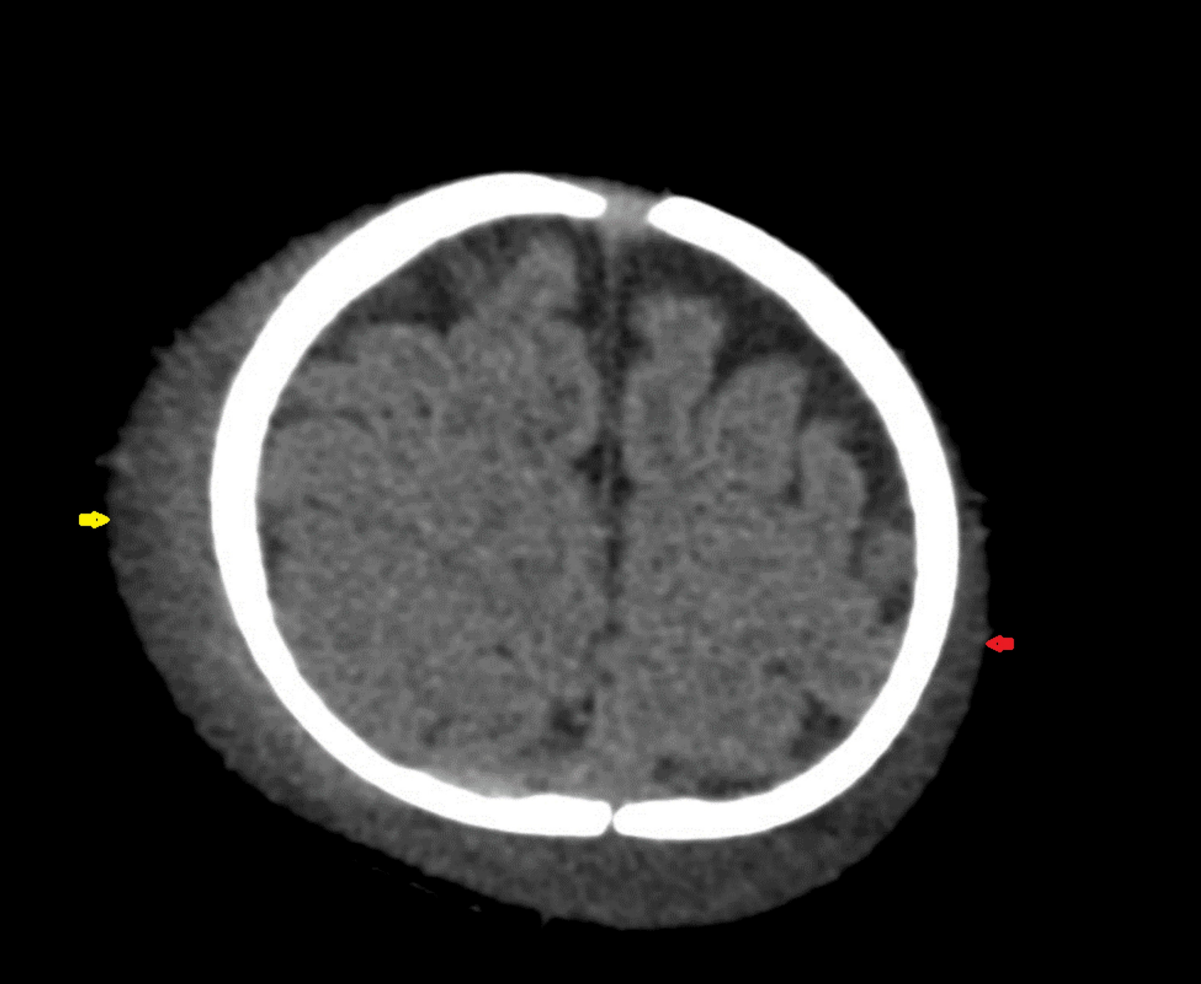
CT of the brain (axial plane) showing right-sided subgaleal hematoma (yellow arrow) with involvement of the opposite side (red arrow).

**Figure 4:**
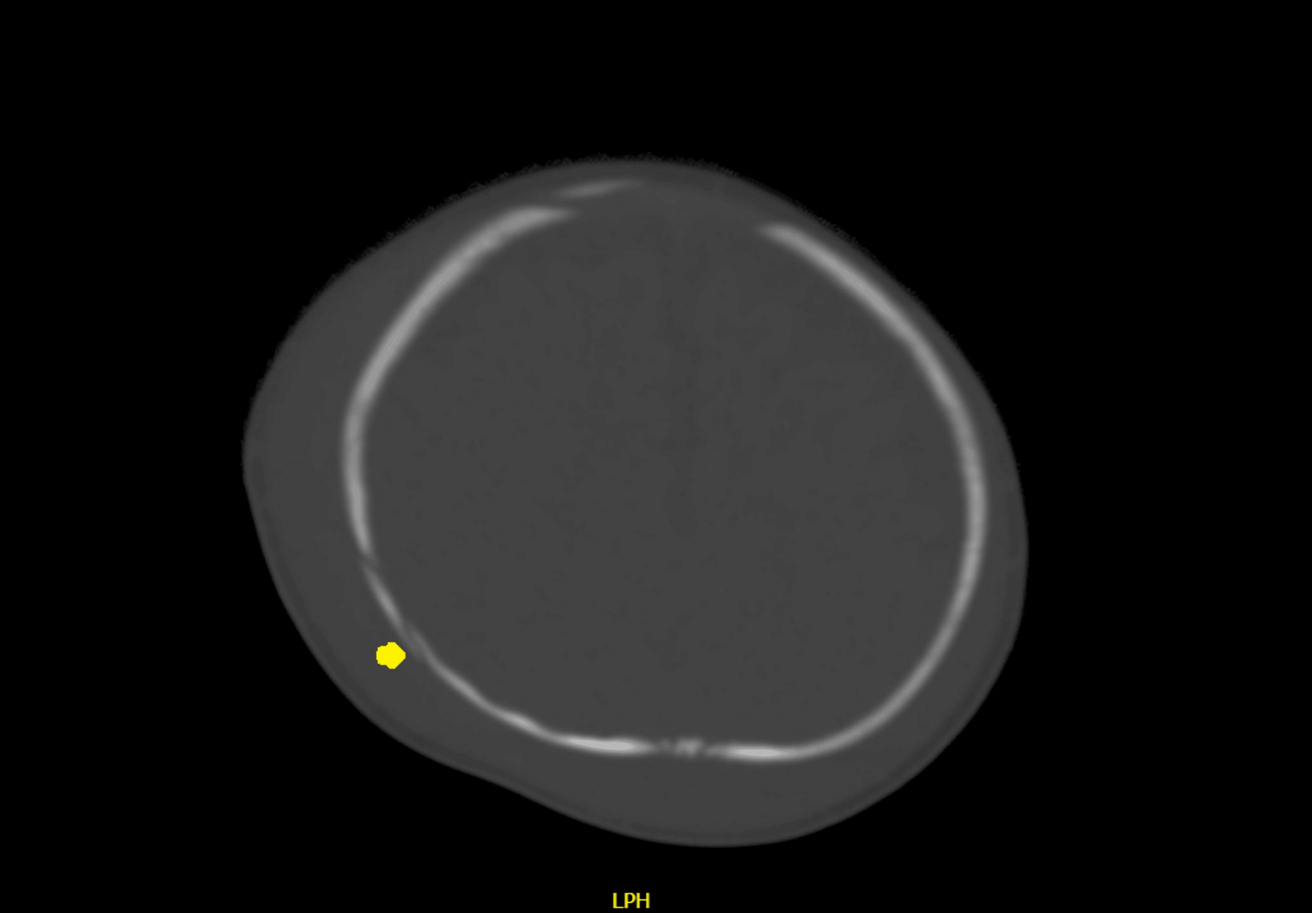
CT of the brain (axial plane) showing fracture of the right parietal bone.

CT of the brain showed fracture of the right parietal bone, with subgaleal hematoma with a maximum thickness of approximately 11 mm (Figures 1-4). However, the subgaleal hematoma was overlying almost the entire right hemi cranium with a small extension to the contralateral side. No extra-axial or intracerebral areas of fresh blood density were observed.

The patient was hospitalized for four days and received intravenous (IV) antibiotics, including IV ceftriaxone and IV vancomycin. Clinical improvement was noted by the fourth day of admission, with the patient becoming afebrile, demonstrating improved activity, and maintaining good oral intake.

## Discussion

Subgaleal hematoma is a complication following head trauma that usually occurs in children. It typically appears as a fluctuating mass that gradually increases in size. Subgaleal hematoma is uncommon, and infection of subgaleal hematoma is extremely rare, especially if the skin barrier is intact.

Here, we presented the case of a five-month-old male infant with a history of blunt head trauma. He had a later worsening of his clinical condition, signs of local infection (skin erythema, local warmth, and tenderness), and very high inflammatory markers. The clinical findings were consistent with an infected subgaleal hematoma.

Due to its rarity, there are no standard guidelines for the management of infected subgaleal hematoma. Needle aspiration can be both diagnostic and therapeutic, and along with antibiotics, it plays an essential role in management. Surgical intervention and drain placement have been performed in cases complicated by abscess formation and skull osteomyelitis [17-20]. Neurosurgical consultation must be sought in these situations.

## Conclusions

Infection of subgaleal hematoma after blunt head trauma is an extremely rare complication that should be considered in pediatric cases that develop fever after head trauma associated with signs of local infection, increasing hematoma size, and irritability. Early treatment with an appropriate antibiotic regimen can help prevent complications such as subgaleal abscess and skull osteomyelitis.
